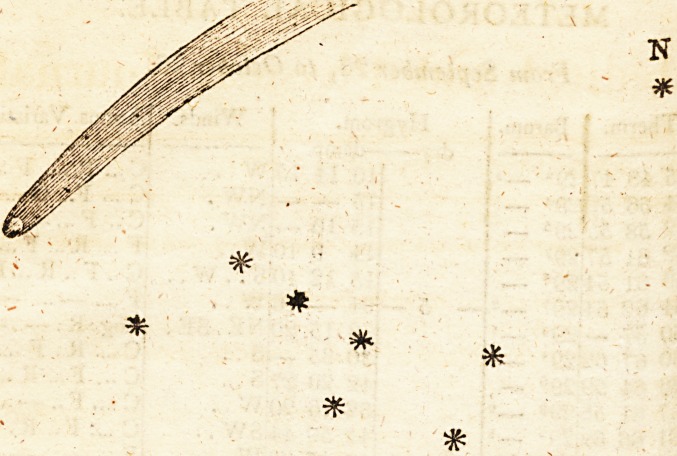# Meteorological Table

**Published:** 1811-11

**Authors:** 


### METEOROLOGICAL TABLE.

### *From September 26, to October 26.*

| D | Therm. | | | Barom. | | Hygrom. | | | | Winds. | Atmos. Variation. |
|---|---|---|---|---|---|---|---|---|---|---|---|
| | | | | | | dry——damp | | | | | |
| 27 | 45 | 48 | 47 | $29^{2}$ | —$^{3}$ | | 10 | 14 | 15 | W .. | C ... R .... F ... |
| 28 | 44 | 56 | 52 | $29^{4}$ | —$^{5}$ | | 15 | — | — | NW . | C ... F... C ... R. |
| 29 | 52 | 58 | 55 | $29^{6}$ | —$^{7}$ | | 15 | 10 | — | NW . | C .. F ... — .... |
| 30 | 52 | 61 | 57 | $29^{7}$ | — | | 14 | 9 | 10 | W .. | F .... R ... F .... |
| 1 | 59 | 61 | 54 | $29^{6}$ | — | | 15 | 12 | 10 | S . . W . . | C .. F .. R ... F ... |
| ◐ 2 | 54 | 62 | 54 | $29^{7}$ | —$^{8}$ | — 5 — | 84 | — | 6 | W . . | F .... — .... — .... |
| 3 | 50 | 57 | — | $29^{9}$ | —$^{5}$ | | 8 | 15 | 20 | NE . SE . | Fog . R . — .. — .. |
| 4 | 60 | 67 | 62 | $29^{5}$ | — | | 30 | 35 | — | S . . | C .... R . F .... |
| 5 | 66 | 64 | 59 | $29^{6}$ | — | | 42 | 20 | 27 | S . . | C ... F ... R ... |
| 6 | 57 | 61 | 58 | $29^{8}$ | —$^{9}$ | | 32 | 16 | 20 | W . . | C .... F .. — .... |
| 7 | 61 | 66 | 62 | $29^{9}$ | —$^{8}$ | | 45 | 50 | 44 | SW . . | C .... F .. R . C ... |
| 8 | 60 | 61 | 59 | $29^{9}$ | 30 | | 48 | 15 | 23 | W . . | F ... — ... — ... |
| ○ 9 | 61 | 66 | 62 | 30 | — | | 35 | 32 | 33 | W .. | R . F . C ... |
| 10 | 59 | — | 61 | 30 | — | | 35 | 33 | 35 | W . | C .... — ... R .. |
| 11 | 61 | 66 | 62 | $29^{9}$ | —$^{8}$ | | 38 | 30 | 36 | W . . | C .... F .. C ... R. |
| 12 | 57 | 62 | 56 | $29^{8}$ | —$^{7}$ | | 32 | 34 | 33 | S . . SW . . | C ... F .. R ... |
| 13 | 52 | 61 | 55 | $29^{8}$ | —$^{9}$ | | 30 | 1 | 11 | W . . . | F ... — ... C ... |
| 14 | 55 | 62 | 60 | $29^{9}$ | — | | 20 | 43 | 41 | SW . . | R .. C .... F ... |
| 15 | 60 | 70 | 65 | $29^{9}$ | —$^{8}$ | | 39 | 14 | 19 | S . | F .. — .... — .... |
| 16 | 62 | 63 | 64 | $29^{8}$ | 30 | | 26 | 20 | 25 | SW . . | F .... C .. R . F .. |
| ◑ 17 | 63 | 68 | 62 | 30 | $30^{1}$ | | 27 | 24 | 27 | W . | C .. F . . — .... |
| 18 | 57 | 67 | 63 | $30^{1}$ | —$^{2}$ | | 20 | — | 27 | SW . . | Fog... F.... R.... |
| 19 | 61 | 65 | 58 | $30^{2}$ | — | | 32 | 36 | 30 | W . | R.. F...C..Fog.... |
| 20 | 58 | 63 | 59 | $30^{2}$ | —$^{1}$ | | 32 | 30 | — | SW . . | C . F ... C ... |
| 21 | 58 | 64 | 62 | 30 | $29^{7}$ | | 30 | 25 | 32 | W . . | C .. F ... C ... |
| 22 | 62 | 64 | 56 | $29^{5}$ | — | | 33 | 30 | 32 | SW . . | C .. R .. — ... |
| 23 | 54 | 58 | 54 | $29^{6}$ | — | | 30 | 16 | 23 | W . . | F .. — .. — ... |
| 24 | 54 | 55 | 54 | $29^{5}$ | —$^{4}$ | | 28 | 25 | 26 | SW . | C .. R .. C .. |
| ● 25 | 49 | 54 | 48 | $29^{4}$ | —$^{1}$ | | 24 | 2 | 18 | W . . | F.... R.. R... in N. |
| 26 | 49 | 50 | 48 | $28^{8}$ | — | | 23 | 20 | 23 | SW . . | F ... R ... — .. |

Quantity of rain from September 26 to October 26, two inches and $\frac{5}{100}$.

The temperature of this interval has generally been considered as unusually great; but it will be seen, by referring to the registers of last year, that a very small difference has existed between the heat of October 1810, and 1811. In 1810 the greatest height of the therm. in the middle of the day, on the 8th, was 71; in 1811 the greatest heat was on the 15th, when the therm. in the middle of the day was 70. The lowest point in J810 was 50, in 1811 also 50. The quality of the heat of the present October has, however, been peculiarly oppressive, and this has arisen possibly from the great humidity of the atmosphere, which has, at times, occasioned the metropolis to be involved in a vapour bath.

The most interesting meteorological occurrence of this month, as of the last, is the Comet. As the moon ceased to give light in the evening this orb became very conspicuous, and early in the month shone with considerable splendour. At eight o'clock in the evening of the 6th it was particularly distinct, and, perhaps, better defined than at any other period. It was seen to the west of the great Bear, illumining all that region, and extending its *coma* over an immense space. Its apparent position, and relative magnitude, as observed on the evening of the 6th, will be understood by the annexed diagram.

On

On the 9th of September, the time of the perihelion passage of this Comet, it was distant from the Sun 94,724,260 miles. On the 13th of September, its distance from the Earth was 142,500,000 miles. On the 15th its distance from the Sun was 95,258,840 miles; the distance of the Earth from the Sun, at that time, being 95,505,923 miles. The length of the Coma 33,000,000 miles. The motion of this Comet is from E. to W. being the reverse of what it appears to a spectator on this earth. Its real size, as deduced from its appearance in the grand Herschelian telescope, is about that of our moon: the brilliant central nucleus is invisible in the 10 feet Herschelian, and in every smaller telescope. This statement, determined on the formula of the Mechanique Celeste of la Place, has at least an air of precision.

Opinions respecting the nature, qualities, and influence of Comets, have generally been portentous. We have before slightly observed on the popular notions; nor does it seem that philosophy has been altogether free from conclusions improbable if not absurd. Sir Isasc Newton (*Principia*) calculated that the Comet of 1680, when in its perihelion, was to the mean distance of the Earth to the Sun, in the ratio of about 6 to 100; from whence it was inferred that its heat in that part of its period should have been to our summer heat reciprocally as the squares of their numbers, that is, as 1,000,000 to 36, or 28,000 to 1; and on this data he computes the heat of that Comet to have been 2000 times greater than red hot iron. But of what matter must this orb have been made to be susceptible of such a prodigious and terrible heat? If Comets are in their nature like our Earth, and have an atmosphere similar to ours, they are incapable of being heated to that enormous degree.

*Princes Street, Cavendish Square.*

## Figures and Tables

**Figure f1:**